# Characterization and Application of Calotropis Procera Fiber as a Sustainable Filter for Oil Removal from Aqueous Emulsion

**DOI:** 10.1021/acsomega.5c00525

**Published:** 2025-05-05

**Authors:** Eduardo Perini Muniz, Lucas Prandi Coutinho, Odilon Junio Gonçalves de Oliveira, Marla Almeida Siqueira, Paulo Sérgio da Silva Porto, Edson Caetano Passamani, José Rafael Capua Proveti, Cleocir José Dalmaschio

**Affiliations:** † Programa de Pós-Graduação em Energia, 28126 Universidade Federal do Espírito Santo, Rodovia Governador Mario Covas, km 60, Bairro Litorâneo, São Mateus, Espírito Santo 29932-540, Brazil; ‡ LabPetro - Programa de Pós-Graduação em Química, Universidade Federal do Espírito Santo, Av. Fernando Ferrari, 514—Goiabeiras, Vitória, Espírito Santo 29075-910, Brazil; § Programa de Pós-Graduação em Física, Universidade Federal do Espírito Santo, Av. Fernando Ferrari, 514—Goiabeiras, Vitória, Espírito Santo 29075-910, Brazil

## Abstract

Fibers extracted from Calotropis procera (CP) fruits were successfully used to filter engine oil from a synthetic oil-in-water effluent. Fiber morphology was examined by scanning electron (SEM) and optical (OM) microscopies, while their structural, vibrational and chemical properties were systematically studied by thermogravimetric analysis (TG), X-ray diffraction (XRD), and Fourier transform infrared (FTIR) spectroscopy. It was first demonstrated that the fibers are tubular with strong anisotropy and that when placed in oil, most oil adsorption occurs at their internal surfaces, a region with higher hemicellulose concentration. Contact angle experiments and standard gravimetric measurements confirmed that the fibers are hydrophobic, while FTIR results have suggested a small amount of water retained within the fiber walls after forced submersion. Using a cylindrical filter in continuous mode and a semispherical filter in batch mode, the percentile of oil removal (OR %) was 67% for 200 mg of fiber mass (FM) and a flow rate (Q) of 172 mL min^–1^ and higher than 95% when the effluent is under agitation at 140 rpm and FM of 30 mg. The OR % variation data with FM in the cylindrical filter was consistent with a constant removal ratio as a function of penetration depth, whereas the results for the semispherical geometry have indicated an increase in OR % with effluent speed. The fibers retained their effectiveness throughout three reuse cycles, consistently adsorbing more than 50% of the emulsified oil. Thus, this study represents the first report on the application of CP fibers as an oil filter for oil-in-water emulsions and provides the most detailed chemical composition analysis of these fibers to date.

## Graphical abstract

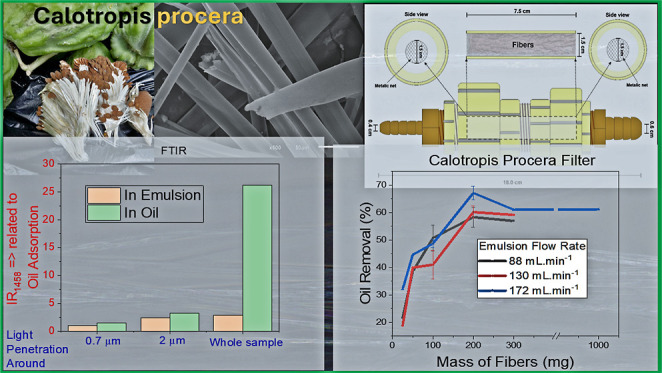

## 1 Introduction

It is well-established that mineral oils are a necessary agent in machinery to reduce friction and heat. However, these petroleum compounds show, in general, low biodegradability, often leading to environmental contamination.^1^ Thus, they disrupt significantly aquatic ecosystems by forming a thick surface film on effluents, a condition that reduces, for example, the oxygen exchange between the water and the external atmosphere, affecting the water oxygenation process. Mineral oils also can interact with the environment, producing ecotoxic secondary products that may prove even more detrimental than their original chemical forms.^2^ Besides that, the emulsified oil frequently increases water turbidity leading also to the death of water flora by lack of sunlight.

To address these issues, several processes are currently being applied, but new materials and modified methods should be still tested. For example, gravitational settling tanks are commonly employed to separate oil from water, a method favored by small businesses (e.g., gas stations) due to its simplicity and efficiency in removing most floating oils. This widespread technique has influenced several legislations around the world. In Brazil, for instance, the law permits a discharge up to 100 mg L^–1^ of oil into the sewers, favoring a condition for absence of a thin layer of visible floating oil after oil discharge.^3^ However, as oil travels through pipelines and interacts with impurities, it can form stable emulsions that bypass the settling tank. To reduce this problem, some settling tanks include a coupled activated carbon filter, a technically viable solution. Many business owners consider activated carbon costly though, and the spent filtering cartridge is a solid residue that needs proper treatment.

Despite the perceived costs, filtration offers advantages for small businesses, including simplicity, space efficiency, and ease of operation without needing specialized labor. In this regard, the scientific literature and commercial guides describe a large selection of filtration materials. In principle, there are two types of materials for filtration, i.e., those that are oleophilic and hydrophobic to retain water, but allow oil passage^4^ or those that are hydrophilic and oleophobic to perform the inverse process.^5,6^ Among the filter materials, a possibility still not fully explored is the use of local plants found in several countries. A cost-effective solution would be oleophilic and hydrophobic natural fibers readily available in tropical regions and that do not require any treatment or membrane fabrication before their use. Here, we would like to stress that there are several natural fibers suitable for this type of application, i.e., cellulose-based fibers (e.g., Kapok),^7–9^ Calotropis gigantea,^10–12^ and Calotropis procera (CP),^13–15^ natural materials that have gained attention for their ability to effectively adsorb oils.

Specifically, CP is a drought-resistant, salt-tolerant species from the Apocynaceae family extensively documented in the literature because of their myriad uses and broad geographical distribution, particularly in tropical regions.^16^ Indeed, this species is commonly found in arid or semiarid regions across the Middle East, Northern Africa, South America, and India, where it is also recognized for its medicinal properties.^17^ Additionally, CP has applications in veterinary medicine care,^18^ serves as livestock forage,^19^ and its latex-derived cysteine peptidases are employed in cheesemaking.^20^ CP leaves have also been evaluated as a coagulant-flocculant agent for water treatment,^21^ whereas fibers extracted from CP fruits have been the subject of studies exploring their effectiveness as oil adsorbers for oil spill cleanup.^14^ The fibers extracted from CP fruits are hollow cylindrical tubes covered in hydrophobic wax,^13,22^ a surface layer responsible for the fiber hydrophobicity. On the other hand, while, several studies have indicated that the CP fibers are highly oleophilic^13–15^ there is no report studying systematically the use of CP fibers in near conventional geometry of pipelines to filter emulsified oils. Furthermore, the studies reporting the adsorption properties of CP fibers^13–15^ do not describe how the adsorbed oils are distributed on the fiber external and internal walls.

Therefore, this work addresses these two main gaps by analyzing a simple yet effective system that could be adapted in ordinary pipelines to reduce environmental contamination from oil in aqueous emulsions. The chemical characterization and the performance of CP fibers as oil adsorbers were systematically investigated, particularly in removing emulsified oils from water. The tests were done after the CP fibers had been carefully arranged within a cylindrical tube with a flow of oil-contaminated effluent to simulate the operation of an ordinary pipeline. The feasibility of reusing these fibers was also tested applying mechanical compression to recover oil between the filtration cycles. Besides the adsorption tests, Fourier transform infrared spectroscopy (FTIR) was employed to investigate the possible chemical paths for the oil adsorption process, while a fundamental characterization of structure and morphology of the CP fibers was performed using thermogravimetric analysis (TGA), X-ray diffraction (XRD), optical microscopy (OM), and scanning electron microscopy (SEM). Thus, our results constitute a step forward in the use and characterization of the CP fibers to filter the emulsified oil flowing in ordinary-like pipelines.

## 2 Methodology

### 2.1 Calotropis procera Fibers

The fruits of CP, like those shown in Figure 1a, were harvested in the Conceição da Barra municipality (State of Espírito Santo/Brazil) in December of 2022. After fruit collection, the CP fibers, Figure 1b, were manually separated and were dried at room temperature (RT) for 24 h, to be finally stored in a plastic container for later use. The only processing performed on the fibers was the removal of their seeds and leaving them for 1 day resting over a table under ambient conditions to reach a dry-like state.

**Figure 1:**
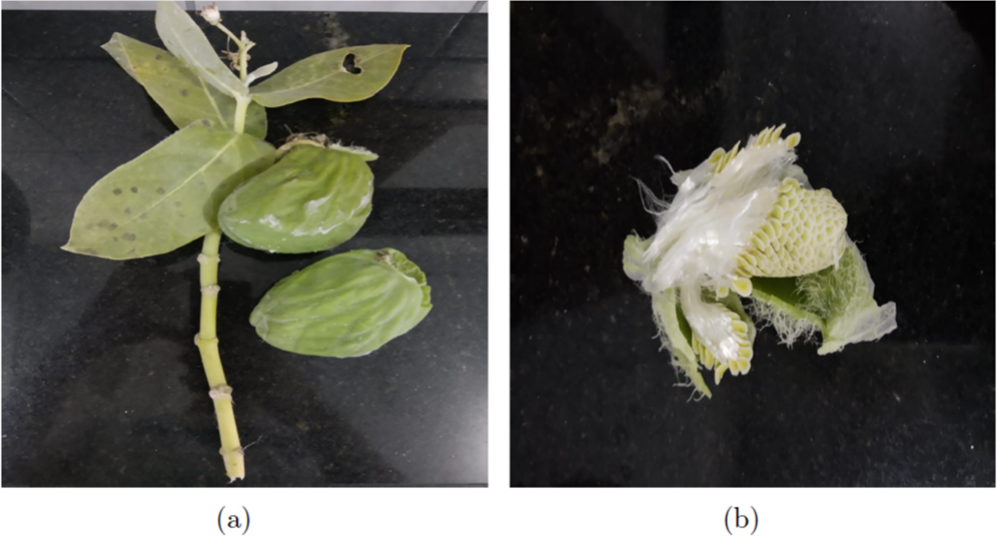
Parts of Calotropis procera tree used in this study, showcasing the natural morphology of the plant’s fibrous material. (a) Fruit of Calotropis procera and (b) its fibers attached to seeds.

### 2.2 Sample Characterization

#### 2.2.1 Fourier Transform Infrared Spectroscopy

FTIR spectra were measured with a Cary 630 Agilent spectrometer using a resolution of 2 cm^–1^ and 250 scans. Three accessories were used: a germanium crystal attenuated total reflectance (ATRG), a diamond attenuated total reflectance (ATRD), and a Dial Path transmittance accessory (DPT) adjusted for an optical path of 30 μm. The ATRG spectra were assumed to be dominated by the fibers’ surface (≈0.7 μm), ATRD tends to include contributions from greater depths (≈2 μm), and the full width of the sample determined the spectra measured using DPT.

#### 2.2.2 X-ray Diffraction

XRD diffractograms were recorded at RT using a Rigaku Miniflex 600 diffractometer, operating with a Cu Kα radiation source (1.5418 Å wavelength) for 2θ from 10° to 60° in a step of 0.01° at a speed of 2.0° min^–1^. The sample was mounted with the X-ray beam direction projection parallel and perpendicular to the fiber length.

#### 2.2.3 Optical and Scanning Electron Microscopy

Optical microscopy images were acquired using a Leica EZ4HD microscope equipped with a digital camera and configured to a magnification of 350×. The sample was meticulously mounted on a clean glass microscope slide and gently flattened to ensure stability, optimal focus, and clarity during observation. Illumination was adjusted using the microscope’s integrated LED array to provide uniform lighting across the entire field of view. The two movies showing the reaction of CP fibers to water and air were recorded using an USB microscope with magnification from 500 to 2000×.

SEM images were acquired using a JEOL JSM-6610LV microscope operated at an accelerating voltage of 20 kV. The instrument provides a resolution limit of 15 nm. Prior to the experiment, the sample was mounted onto a carbon tape and subsequently sputter-coated with a thin layer of gold to present electrical conductivity and minimize charging effects during analysis. SEM images were typically captured at a working distance of 10 mm and the magnifications ranging from 500× to 10,000×.

#### 2.2.4 Thermogravimetric Analysis

TTGA data were obtained in synthetic air at a flow rate of 10 mL min^–1^. The heating ratio was 10 °C min^–1^, and the initial mass (*m*_0_) was 6.4996 mg. For simplicity, the data will be presented as a function of the percentile of remaining mass (RM %), given by eq 1.

1 $$RM\;\%=\frac{m\left(T\right)}{m_{0}}\times 100$$

where *m*(*T*) is the mass measured at a given temperature (*T*). The thermogravimetric derivative (DTG) curve was obtained by differentiating RM % relative to *T* and using percentile smoothing at 43% in the Origin software. The degree of smoothing was chosen to eliminate a few bad data points while not changing the shape of the DTG curve.

### 2.3 Synthetic Effluent (Emulsion)

A stable oil in water emulsion was prepared mixing 200 mg of HAVOLINE 15W40 semisynthetic lubricating oil (with a density of 0.879 g cm^–3^ at 20 °C and a viscosity of 106.6 cSt at 40 °C) and 20 mg of the emulsifier TWEEN 80 in 1 L of distilled water. The reagents were thoroughly blended using an Ultra Turrax agitator, with the IKA brand model T50 running at a rotation speed of 8600 rpm. For each day of the experiments, 4 L of emulsion was prepared by stirring for three cycles, each lasting 7 min, and with 7 min intervals in between.

Previously published stability tests^23^ indicated that the chosen oil concentration stabilizes at (145 ± 5) mg L^–1^ approximately 12 h after its production and, it has also remained at this concentration for a minimum period of 36 h. Therefore, in this work, all experiments were carried out using optimum emulsions prepared within 12 to 18 h before their uses.

### 2.4 Adsorption Tests for Pure Oil and Distilled Water

The oil adsorption procedures were based on ASTM F726-17.^24^ In the tests, 30 mg of CP fibers were kept in oil for 5 min to 24 h, with, at least, triplicate samples for each time. A “dripping time” of 5 min was adopted before the final measurement of mass. These procedures were also used to determine water adsorption and total adsorption in emulsion. The fibers’ sorption capacity (SC) was calculated via eq 2.

2 $$SC=\frac{W_{t}-W_{t0}}{W_{t0}}$$

where *W*_t_ and *W*_t0_ parameters are the weights of the fibers (in mg) after and before adsorption, respectively.

The contact angle of water on a bed of C. procera fibers was determined using images captured with a 12 MP smartphone camera and analyzed with ImageJ software.^25^ To ensure optimal image capture, the sample was arranged as a bed on a glass microscope slide and carefully adjusted to achieve the flattest possible surface. A 20 μL droplet of deionized water was then deposited onto this surface, and the image was subsequently captured.

### 2.5 Oil Removal in Emulsions

The methodology to determine the oil concentration in water consisted of extracting the organic phase using hexane as the solvent, as adapted from the colorimetric method 5520 of the Standard Methods for Examination of Water and Wastewater.^26^ The oil concentration was determined through UV–Vis measurements at a wavelength of 270 nm of the hexane containing the extracted oil. Equation 3 was used for the determination of the percentile of oil removal.

3 $$\text{OR}\;\%=\frac{C_{i}-C_{f}}{C_{i}}\times 100$$

where *C*_i_ is the oil concentration before and *C*_f_ after the effluent flowed through the filter.

### 2.6 Filtration

A filter column with an internal length of 15.0 cm and an internal diameter of 1.5 cm was used (Figure 2). This filter was connected to a pulse pump with a maximum flow rate of 190 mL min^–1^, controlled by an electronic system. The interior of this column was filled with CP fibers, forming a cylindrical mesh with a diameter like that of the column. For each experiment, 250 mL of fluid was filtered. Pictures showing the placement of the fibers inside the filtering system are provided in the Supporting Information (Figure S1).

**Figure 2:**
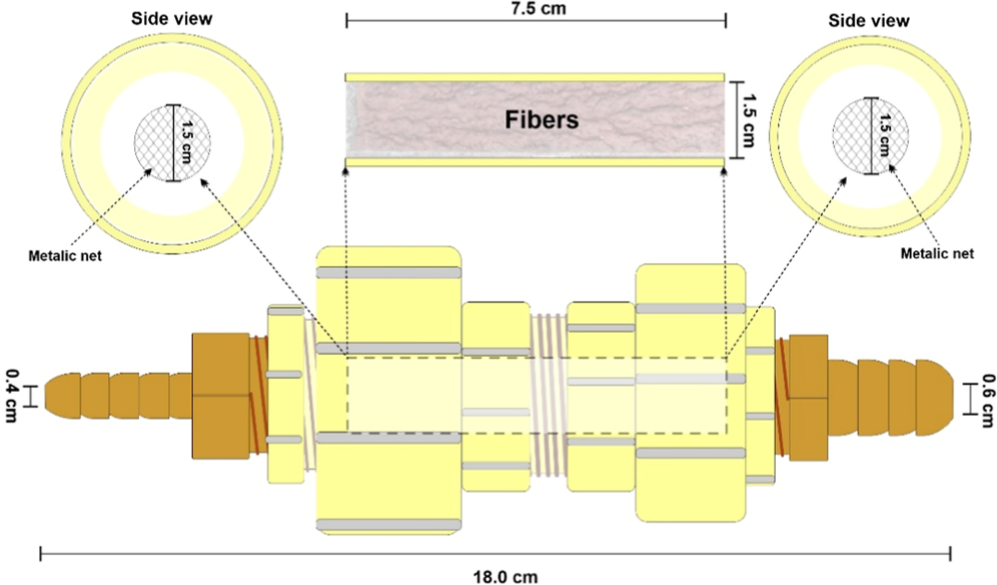
Filter column built to carry out the separation experimental procedure.

To gain insight into the adsorption kinetics, another filtering system was used: 20 mg of fibers were placed under a semispherical metallic net with the same spacing shown in Figure 2 and placed at the bottom of a beaker under 50 mL of emulsion. The system was agitated by an orbital shaker with a stirring speed of either 140 or 50 rpm for 5, 20, or 40 min.

### 2.7 Experimental Design

The preliminary tests have been used 25, 50, and 100 mg of CP fibers. Based on the initial results, a 3^2^ full factorial experimental plan was developed with volumetric flow rate (*Q*) values of 88, 130, and 172 mL min^–1^ (three levels) and values of fiber mass (FM) of 100, 200, and 300 mg. The *Q*-levels were chosen based on the maximum and minimum values supported by the pump, for reliable continuous flow. The FM levels were set based on the highest mass that could be used while maintaining said continuous flow. Statistically, this experimental design demands only a triplicate at the central point to determine uncertainty, considering that the error is aleatory, and that, there is no reason for an experiment to have a standard deviation higher than the others.

Nonetheless, since there is some variation among natural fibers, more experiments than strictly needed for the statistical analysis were performed. The complete set of experimental conditions tested is summarized in Table S1, where the number of tests performed for each combination of variables is noted. In all the experiments, the flow rate stabilized approximately 1 s after initiation, and no noticeable flow reduction was observed due to the presence of the fibers, even in the single experiment where 1000 mg of fibers were used. Origin software^27^ was used to analyze the data.

### 2.8 Fiber Reuse

To expel the adsorbed oil, the fibers were pressed in a hydraulic press by an equivalent weight of (2.0 ± 0.3) Tons. They were confined in a piston consisting of a rod with (2.05 ± 0.01) cm diameter allowed to freely move inside a cylinder with (2.20 ± 0.01) cm of internal diameter. Under this condition, a pressure of (59 ± 8) MPa was uniformly applied on the fibers. After the pressure was applied for about 1 min, the fibers were reinserted into the filtering system and used to treat another 250 mL of effluent. Reusing the fibers for up to 3 consecutive cycles of filtering → pressing → filtering was possible. Under this condition, the fibers broke at the fourth application of pressure.

## 3 Results and Discussion

### 3.1 Fiber Characterization

#### 3.1.1 Morphological and Structural Properties

Results from OM and SEM analyses suggested that the fiber morphologies (see Figure 3) are consistent with what is reported in the literature.^13,22^ The CP fibers are transparent and have diameters ranging from 19 to 36 μm (average value of 26 μm), as shown in Figure 3a inset. In addition, SEM data (Figure 3b) revealed that the surface of the fibers is smooth, except for some nanowhiskers, and that the fibers are hollow tubes with relatively thin walls (see insets in Figures 3b and S3).

**Figure 3:**
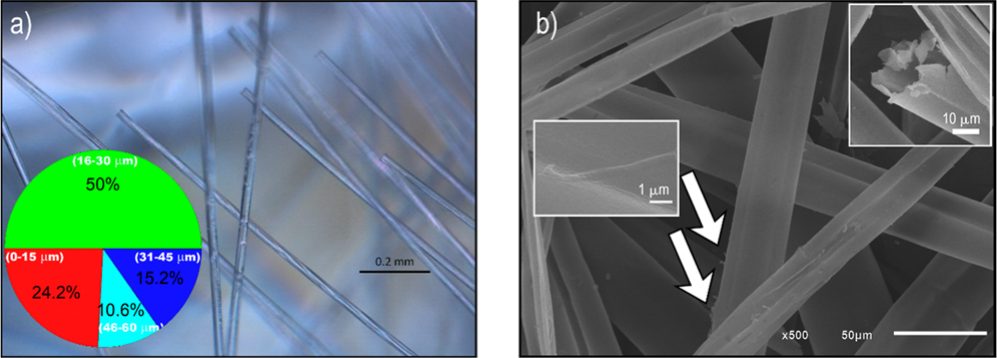
Microscopy characterization of CP fibers: (a) OM image showing fiber morphology and diameter distribution; (b) SEM images highlighting the surface regularity and the tubular fiber structure.

The tubular geometry leads to an anisotropic effect in the XRD diffractograms shown in Figure S4. It can be observed that the XRD diffractogram changes according to the orientation of the main axis of the fiber relative to the direction of the incident X-ray beam. This condition leads to two distinct results: one where the X-ray beam direction projection is parallel to the fiber horizontal plane (Figure S1a) and another where it is perpendicularly aligned to the fiber length (Figure S1b). The assignment, based on the works of French^28^ and by Ahvenainen and co-workers^29^ of the XRD peaks, is displayed in Table S2. The Bragg peaks were fitted considering the method-3 reported by Ahvenainen and co-workers.^29^

The absence of the peak at 2θ = 21°, when the projection of the X-ray light beam is perpendicular to the fiber length (Figure S1b), indicates: (i) the anisotropic effect seen by OM and SEM and (ii) that the crystalline structures in the fibers have a preferential direction of alignment.^28^ The area of the broad peak centered at about 2θ = 16–17° (assigned to amorphous-like phases^43^) also shows a dependence with the X-ray beam direction relative to the fiber length, being smaller for the configuration with the light bean projection parallel to the fiber length, again a feature of the anisotropic-like effect of the fibers.

#### 3.1.2 Thermal, Vibrational and Chemical Properties

To characterize the thermal properties of the fibers, TGA data are presented in Figure 4. The analysis highlights the four main thermal events previously observed for TGA experiments performed under N2 atm reported in the literature for CP fibers^15^ and/or fibers extracted from other parts of the CP tree.^30^ These four events, which occur as the temperature increases, are respectively associated with the loss of moisture and water content, degradation of hemicellulose, degradation of cellulose, and finally degradation of lignin.^15,30^ In our case, the first three events at lower temperatures are similar to those reported in the literature,^15,30^ though there are slight differences in their shapes. For example, the degradation of lignin appears as a sharp decline under air atmosphere instead of the gradual curve measured in the N2-rich atmosphere. This difference can be attributed to the thermal decomposition of lignin at 301 and 428 °C, as also reported for Birchwood in air^31^ (a work also done under a similar heating ratio to that one used in the present work).^32,35–37,39,40^

**Figure 4:**
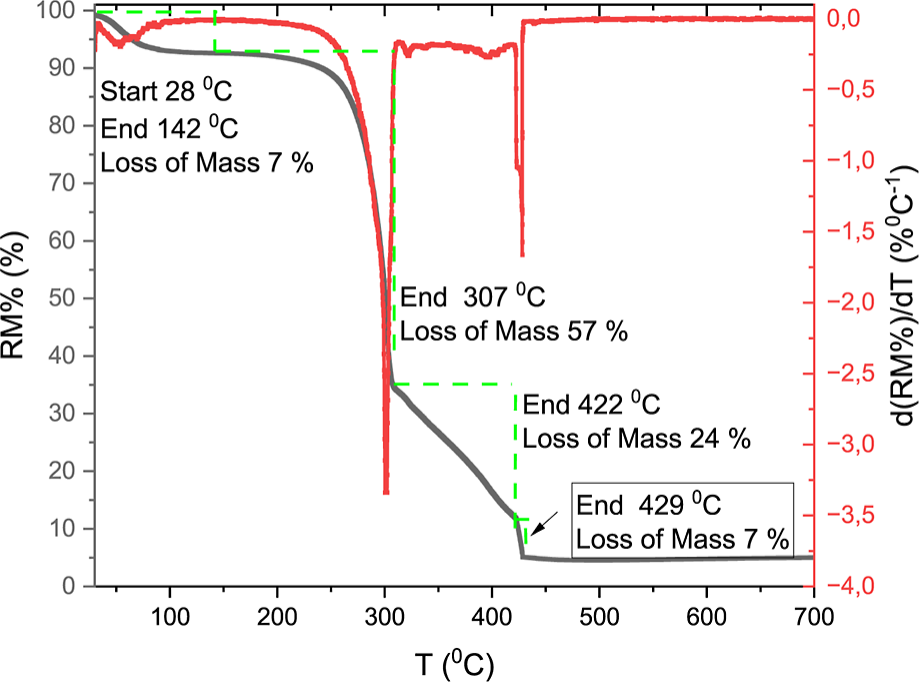
Thermal analysis of Calotropis procera fibers: thermogravimetric analysis (TGA) and derivative thermogravimetry (DTG) curves highlighting four distinct mass loss events, described in terms of temperature range, end temperature, and corresponding mass loss, as represented in the graphic.

As can be deduced from Table S3, there is a remarkable similarity in the moisture content of fibers harvested from different regions, and even from other parts of the plant. The reduction in RM % in the first event indicates no combustion up to 200 °C independently of the atmosphere (air or N2). On the other hand, the reduction in RM % in the second (2nd) event strongly indicates that there is more hemicellulose in the CP fibers extracted from the fruits than those taken from the branches^30^ (see Table S3). This observation will support the oil adsorption mechanism, discussed ahead. For this second-event, only minor differences were observed between the values obtained from CP fibers harvested by different research groups located more than 1000 km away from each other. This reproducibility is also supported by results of TGA and FTIR spectroscopy of the samples harvested in December 2022 and in January 2024 (Figures S5 and S6). These observations are crucial, as they suggest a correlation between hemicellulose content and oil absorption, which will be analyzed in the subsequent section. In a similar vein, the reduction in RM % during the third event remains practically identical across all fiber samples. The comparable cellulose composition between CP fibers from branches^30^ and fruits is particularly noteworthy, given the higher hemicellulose concentration in fruit-derived fibers. The fourth event shows that there is combustion of the lignin above 422 °C. Now, the difference between our results and those reported in the literature^15^ increases to around 3%. However, it can still be inferred that there is more lignin in the fruits than in the branches.^30^

In agreement with the TGA results previously discussed, the FTIR spectra presents bands associated with cellulose, hemicellulose, and lignin (Table S4, Figure 5).

**Figure 5:**
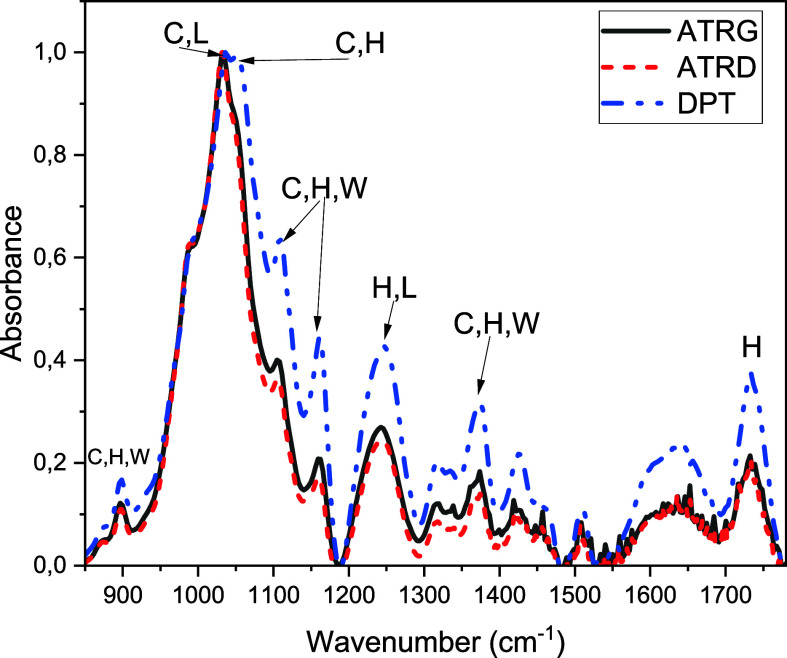
FTIR spectra of CP fibers obtained using three measurement geometries to evaluate differences in chemical group identifications considering different optical paths. Legend: C = cellulose, L = lignin, H = hemicellulose, W = wax.

Supposing a refractive index of approximately 1.5 for CP fibers due to their organic composition, the penetration depth of infrared radiation is estimated at ≈0.7 μm for a germanium crystal and 2 μm for a diamond crystal.^41–43^ These values can be used as a reference for an approximate depth profile of oil adsorption by the CP fibers. Considering the SEM results that indicated that more than 75% of the CP fibers have diameters larger than 15 μm (Figures 3a inset and S3), and comparing to the estimated depth of penetration of each technique, ATRG represents the FTIR spectra of the external surface, ATRD is associated with the spectra of the wall of the tubes and DPT yields the whole fiber spectrum. The main FTIR band associated with the cellulose phase was taken as a reference since it is the most intense in the spectra (Figure 5). FTIR spectra of this material are complex with peak assignments overlapping among multiple components, as shown in Table S4. However, some conclusions may still be reached, providing an approximate profile of the concentration of the components of the fiber as a function of depth relative to the external surface.

Hemicellulose is the easiest component to analyze due to its strong absorption peak at 1732 cm^–1^ (peak at the highest wavenumber in Figure 5). Its relative intensity is higher for DPT than ATRG and ATRD. Other absorption peaks that may be assigned partially to hemicellulose (1051, 1244, 1347, and 1449 cm^–1^) show the same tendency of intensity at DPT > intensity at ATRG > intensity at ATRD. It must be noted that the band at 1051 cm^–1^, associated with either hemicellulose or cellulose, is not easily observable in the ATRG and ATRD spectra, but it forms a second peak with higher intensity in DPT. The combination of these facts leads to the conclusion that hemicellulose is more abundant on both external and internal fiber surfaces than within the fiber wall.

On the other hand, ligniǹs absorption peaks in the FTIR spectra are largely superimposed with those of other components (Table S4), whoever its concentration can be associated with the absorption band at 1508 cm^–1^,^33,34,38^ which has lower intensities at the ATR spectra than in the DPT ones. Therefore, lignin is probably more concentrated in the interior of the fiber, likely in the internal wall since this is the region not fully reached by the ATR measurements.

For cellulose, absorption bands at 1362, 1369, and 1428 cm^–1^ are exclusively associated with this phase and are only discernible in ATR spectra. Moreover, these bands are more intense in the ATRG spectrum than in the ATRD one (Table S4). Conversely, other bands (at 1419 and 2916 cm^–1^) exhibit higher intensities in the DPT spectrum. For the 2850–2963 cm^–1^ region (ν C–H), more distinct bands are seen in the DPT spectrum, implying a greater variety of C–H, CH_2_, or CH_3_ structures. Thus, there is an anisotropic cellulose composition, changing with depth within the fiber.

Finally, some bands associated with waxes were also observed in the FTIR spectra. The absorption bands at 1435 and 1472 cm^–1^, which can be attributed solely to wax, are distinguishable in the ATRD and ATRG spectra, but not in the DPT spectrum, primarily due to the overlap with adjacent bands from other components. The wax-related bands at approximately 720 cm^–1^, attributed to in-plane ν C–H, are absent from the spectra. This absence strongly suggests a deviation from both carnauba and paraffinic waxes. However, determining the exact wax composition was not possible due to the weakness of the absorption bands and/or their overlap with bands from more abundant fiber components.

In summary, FTIR data strongly suggests that hemicellulose is mainly concentrated at the fiber surfaces, lignin tends to be at the internal walls, and cellulosès composition is substantially anisotropic, which is consistent with XRD data. A wax layer, with a composition not defined, probably covers the fiber surface.

### 3.2 Oil Adsorption and Hydrophobicity

First, the contact angle for water on a layer of CP fibers was measured (Figure S7), yielding a value of 132°, which is slightly higher than the 128° reported by Sobral Hilário and co-workers.^15^ However, they still match in the range where the material is considered hydrophobic. On the other hand, the oil affinity is demonstrated by the sequence of pictures in Figure S8. It must be mentioned here that the oil absorption first occurred through the external walls of the fibers, but as will be demonstrated ahead, the internal surface tends to retain more oil.

The results presented in Table 1 confirm the hydrophobicity and high oil affinity of CP fibers. The sorption capacity (SC) calculated using eq 2, for CP immersed for 30 min in water was (1.2 ± 0.2) g g^–1^, whereas when CP was immersed in oil, SC was (80 ± 5) g g^–1^ after 5 min of immersion, remaining in this range for, at least, 24 h. In the emulsion, SC was (12.5 ± 0.5) g g^–1^ after 24 h (Table 1). The sorption in water was higher than that measured by dos Anjos and co-workers (approximately 0.25 g g^–1^ for 40 min of immersion)^13^ but still negligible compared with the oil sorption.

**Table 1:** Adsorption of Oil by CP Fibers

	SC (g.g^–1^)
water	1.2 ± 0.2
mineral Oil	80 ± 5^a^
emulsion (24 h)	12.5 ± 0.5

a Remains constant from 5 min to at least 24 h.

For comparison purposes, first we must highlight that an increase in the capacity of oil adsorption for CP fibers was obtained using thermal treatment,^15^ hydrothermal treatment, submersion in NaOH 0.1 M, or a solution of NaClO_2_.^13^ The highest crude oil adsorption obtained was 124.60 g g^–1^ when CP was subjected to the thermal treatment at 200 °C. However, the price of this increase in oil sorption is higher water sorption, which is a problem if the fiber is expected to be used to filter oil from water.

Nonetheless, the C. gigantea fiber has SC ≈70 g g^–1 11^ for engine oil, kapok fiber has a SC of 60.51 g g^–1^ for lubricating oil^44^ and chestnut fiber shows a SC of 67.62 g g^–1^ for engine oil SAE 10–40 W.^45^ Care must be taken in using these SC numbers as an evaluation of adsorber quality because the experiments were performed using different oils and by distinct methodologies, but the reported values^11,44,45^ demonstrate that the value found for the natural CP fibers of 80 g g^–1^ is competitive, and this natural material can be suitable as oil adsorbent.

Figure 6 shows indirectly that the water (liquid dyed with food coloring to simplify visualization) flows into the CP fiber hollow tube when it is forcibly submerged. However, when the fiber is released in a dry environment, the water is expelled in less than 5 min by one of the fiber extremities. In Movie S1, water repulsion inside the CP fiber is observable, whereas in Movie S2, the fibers in the oil either sink or remain submerged at what appear to be a constant depth due to the presence of air pockets.

**Figure 6:**
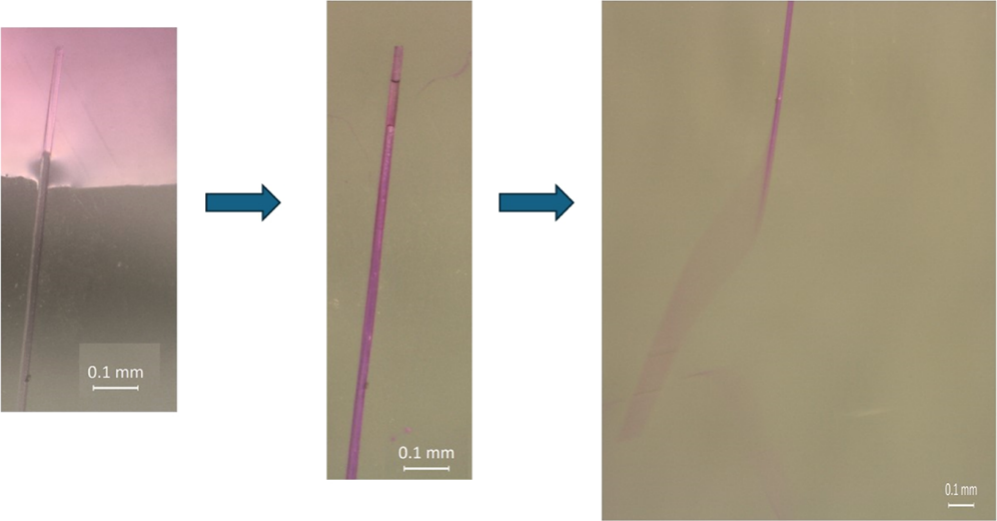
Optical microscopy sequence showing the behavior of Calotropis procera fibers when submerged in water dyed with food coloring, highlighting the interaction between the fibers and the aqueous medium.

Extensive observation by OM led to the conclusion that there is a preferential direction for the movement of water within the fibers, probably in the direction where the seeds were formerly connected to these tubes. A future study of this transport mechanism may be useful for increasing the flow rate inside the fibers, while keeping their oil adsorption ability. Given that the CP fibers are typically found in arid climates and are structurally connected to the seeds, this may explain why they are buoyant and tend to transport water, but do not absorb it significantly.

Movie S2 shows that oil easily enters the interior of the tubular CP fibers. The amount of oil within the fiber cavity after oil immersion is such that the infrared peaks that are usually associated with hydrocarbons due to the νC–H (2800–2950 cm^–1^) have intensities that surpass the scale of our FTIR spectrometer. Therefore, the intensity of the peak at 1458 cm^–1^, the second most intense peak in the spectrum of the oil, was used as an indicator of oil adsorption. To be more precise, the ratio of the intensity of the peaks before and after adsorption (IR_1458_) is represented in Figure 7.

**Figure 7:**
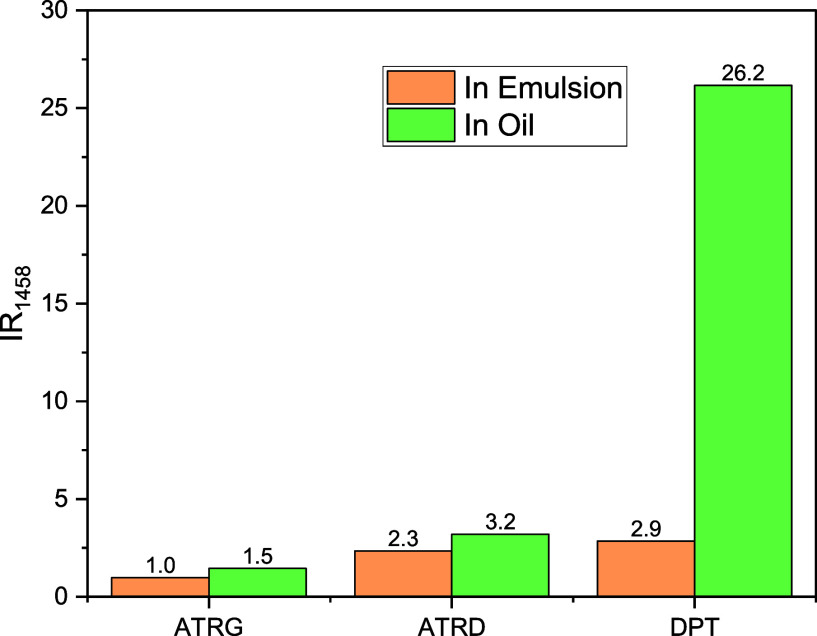
Behavior in the intensity ratio of the FTIR peak at 1458 cm^–1^ when the CP fibers are immersed in oil or in oil-in-water emulsion.

The fact that IR_1458_ in DPT > IR_1458_ in ATRD > IR_1458_ in ATRG, regardless of whether the fibers are in oil or emulsion, demonstrates that the oil tends to be adsorbed at the interior surface of the fibers, followed by the interior of the wall of the tubular fibers (ATRD) (Figure 7) with less oil remaining in the external wall (ATRG). While IR_1458_ in DPT in the emulsion is 126% greater than that in ATRD, in pure oil, it is 818% greater. It is probable that when the water leaves the fiber during the dripping time, it carries part of the oil inside the tube, reducing the difference between the amount of oil imbibed into the wall (related to ATRD) and at the internal surface (related to DPT).

The water distribution in the CP fibers after they were placed in an emulsion and then subsequent dripping for at least, 5 min was ascertained using the δO–H peak due to the adsorbed water (1633 cm^–1^). Figure 8 illustrates the increase in the intensity of this peak when the set of fibers was placed into an oil in water emulsion (IR_1633_). The remaining water is probably adsorbed into the wall of the fibers since the IR_1633_ values in the ATRG and DPT spectra are less than half the IR_1633_ value in the ATRD spectrum. It should be mentioned that by FTIR we do not observe evidence of chemisorption since there is no apparent dislocation of the FTIR bands or alterations in their relative intensities.

**Figure 8:**
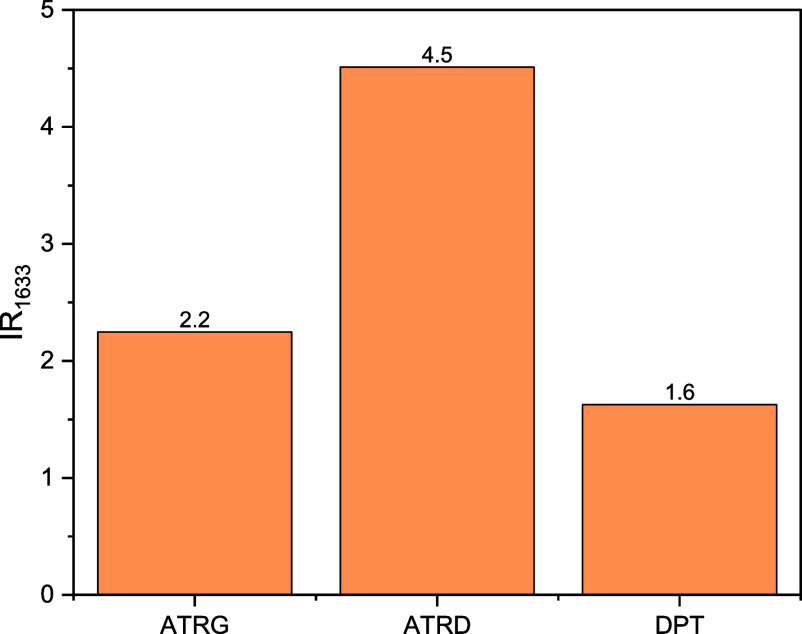
Increase in the intensity of the FTIR peak at 1633 cm^–1^ when the CP fibers are immersed in oil in water emulsion and then left to dry for at least 5 min.

### 3.3 Filtering Oil in Water Emulsion Using CP Fibers

Figure 9 displays OR % by the cylindrical filter as a function of Q and FM. It can be ascertained from this figure that the dominant factor in OR % was FM. This is also confirmed by the Spearman correlation analysis, with a significant correlation of 0.945 between OR % and FM and no significant correlation between Q and OR % (Table S5) and by principal component analysis (Figure S7). Besides that, the lines in Figure 9 represent the fitting of the experimental data by eq 4.

4 $$\text{OR}\;\%=ORf\;\%\;\left(1-e^{-B.FM}\right)$$

where ORf % is the theoretical maximum OR % possible (FM → ∞) and *B* is the ratio of oil removal per unit of FM. Equation 4 can be derived from the considerations done in the work of Davies^46^ (see the Supporting Information) for the retention of particles by fibrous filters in air incorporating the assumptions that (i) there is a maximum limit to OR %, (ii) the volumetric packing factor of the fibers within the filter remains constant, and (iii) the length of the filtrating element increases with FM. Regarding the last two assumptions, the length of the tube was not small enough to compress the fibers, but the diameter was (see Figure S1). The deduction is valid if B is constant along the length of the filter.

**Figure 9:**
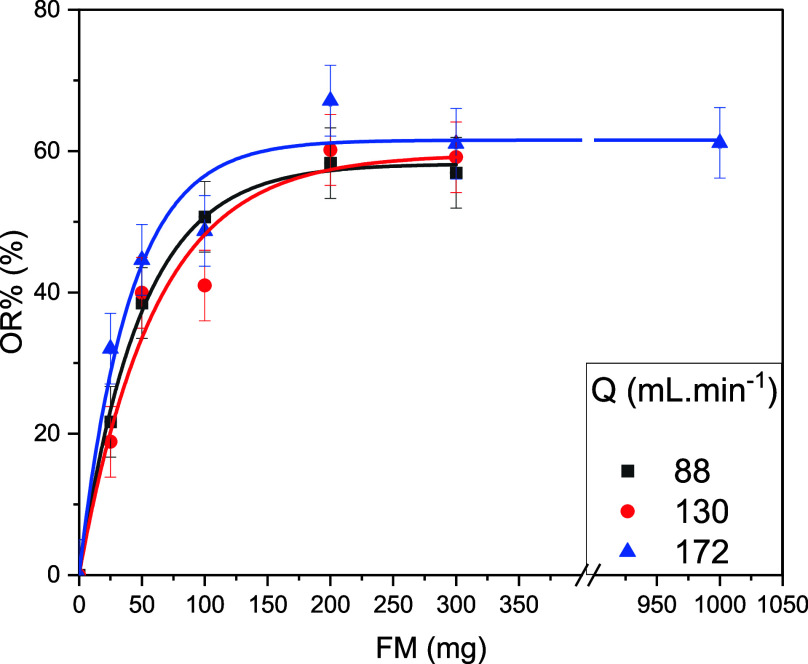
Oil removal efficiency as a function of volumetric flow rate (*Q*) and fiber mass (FM), with the solid lines representing the exponential fittings based on eq 4.

As can be deduced from the fitting coefficients shown in Table 2, *B* and ORF % do not have a significant dependence on *Q*. This implies a constant rate of collision between the oil droplets and the fibers independent of the speed of these oil droplets or, at least, the speed of the fluid that carries them. The average diameter of the oil droplets is between 0.13 and 0.11 μm, as measured in a previous work,^23^ whereas 75% of the CP fibers have diameters greater than 15 μm, and the length of the fibers is between 2 and 4 cm (see OM results). From the perspective of the droplets, the fibers act as broad cylinders, and, although they are open at the extremities, it is less likely that the droplets enter inside the fibers and in contact with their internal surfaces. Oil absorption is most likely related to the collision between the droplets and the external walls of the fibers with oil retention inside the walls as indicated by FTIR spectroscopy.

**Table 2:** Fitting Parameters Used in eq 4 for Modeling the Percentage of Oil Removal by Calotropis procera Fibers

	ORf %		*k*		
*Q* (mL min^–1^)	value	prob > *p*	value	prob > *p*	Adj r-square
88	58 ± 1	<0.001	0.020 ± 0.001	<0.001	0.9709
130	60 ± 4	<0.001	0.017 ± 0.004	0.01	0.9533
172	62 ± 3	<0.001	0.025 ± 0.004	0.002	0.9592

In another set of experiments, it was not possible to measure a consistent OR % when there was no relative movement between the fibers and the fluid. The measurements return values from 57 to 12% for 18 h of immersion, with the results being heavily dependent on how the fibers are forced to sink and how the open sides of the fibers are placed. Therefore, the independence of OR % with *Q* is not absolute. To further demonstrate this point, the fibers were placed under a semispherical net at the bottom of a beaker under agitation. For this system it was possible to study reliably the evolution of OR % with time (kinetics) with a convergence to a maximum value in less than 20 min. The results (Figure 10) show that in this semispherical filter, the fibers can remove more than 95% of the oil, but OR % depends on the rotational speed (RS).

**Figure 10:**
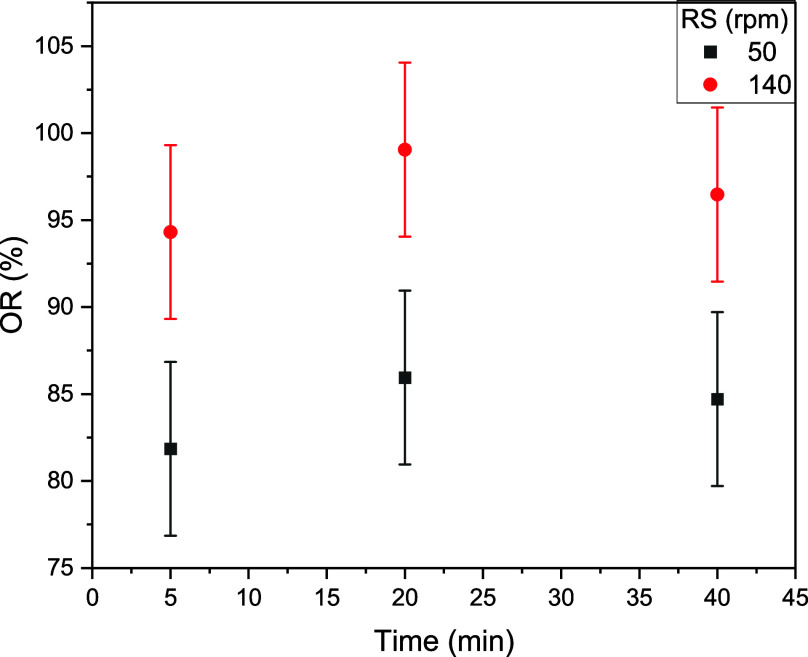
Oil absorption of 30 mg of Calotropis procera fibers placed under a net at the bottom of a recipient with 50 mL of emulsion.

If the oil droplets enter the tubular fibers, the probability of adsorption increases, as demonstrated by FTIR spectroscopy. However, the results shown in Figure 10 imply that if they instead hit the long cylindrical fibers laterally, as probably happens in the semispherical geometry, the amount of oil retained is proportional to the collision speed.

In summary, FTIR spectroscopy demonstrated that the exterior walls of the fibers are less prone to oil retention. However, the results for the filtration systems show that if the droplets collide with walls with enough kinetic energy, they will percolate. The independence of the results with respect to *Q* for the cylindrical filter may be attributed to a variation in droplet speed when moving amid the fibers. In addition, the analysis of Figure 10 reveals a slight increase in OR % with *Q* (although within error the increase occurs for all values of FM).

Singh and co-workers^12^ reported removal efficiencies exceeding 95% using *Q* of 2 mL min^–1^ and a cylindrical filtration system similar to the one in this work. Therefore, there may be a dependence of OR % with *Q* outside the range studied. However, it is unclear if their values can be directly compared to our OR % due to methodological differences, including oil density and concentration. Furthermore, they used Calotropis Gigantea fibers, which appear to have lower oil adsorption than CP fibers in batch mode (pure oil) but have similar diameters. In this regard, Huang and Lim^9^ obtained more than 99% efficiency in oil removal using kapok fibers (that have an external diameter of (17 ± 2) μm) under a pressure of 12.57 kPa using a filter column with 10 cm of length. This condition corresponds roughly to the experimental condition used in our work when the FM reaches values higher than 1 g. The same can be said for the work by Knapik and Stopa^47^ that used sunflower fiber (extracted from the sunflower pith) in a filtration column of, at least, 10 cm length, FM ca. 1.84 g, and Q from 5 to 30 mL min^–1^, obtaining oil removal close to 100%.

In other words, with a low enough *Q* and a long enough filter, higher values of OR % will be achieved. However, it must be noted again that the methods for measurement of oil in water, the type of oils, and their concentrations differ between scientific reports (the results obtained for different ranges of FM, *Q*, natural fibers, and length of the filtering element are complementary). When it comes to filtration technology, the primary contribution of this study, beyond being the first to employ pure and unmodified CP fibers for mineral oil removal from emulsions in a filtration process, is demonstrating that high Q values can still yield significant OR %.

### 3.4 Reuse

As shown in Figure 11, OR % for the cylindrical filter was still above 50%, even after an external pressure of (58 ± 8) MPa was applied to the fibers in three consecutive cycles. Besides the possibility of reuse, the resistance to the three compressions indicates that CP does not have the fragility often attributed as one of the disadvantages of natural fibers.^45^ Some fibers besides CP also show remarkable mechanical resistance, for example, the report on C. gigantea that could withstand 185 kPa with no changes in its structure.^10^

**Figure 11:**
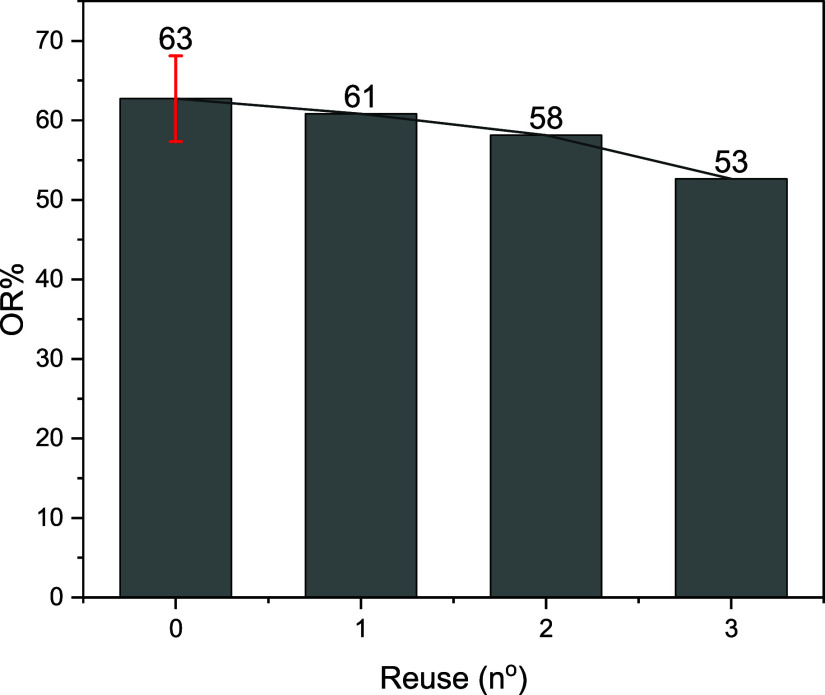
Oil removal efficiency (OR %) after multiple reuse cycles, using a Calotropis procera fiber mass (FM) of 200 mg and a volumetric flow rate (*Q*) of 172 mL·min^–1^.

The reuse after pressure application shows that the cellulosic tubes that comprise the fibers are elastic, probably returning to their original shape after the pressure is released. As can be seen from the TG results (Figure 4), CP fibers are also resistant to heat up to 200 °C; this fact and this newfound mechanical robustness highlight their suitability for various industrial applications.

## 4 Conclusions

This study marks the initial and successful application of untreated C. procera fibers for extracting mineral oils from emulsions. Initially, the natural fibers were systematically characterized to determine their structural (X-ray), morphological (microscopy), thermal (TG), and chemical (FTIR) properties, including the pioneering use of FTIR spectroscopy to obtain an approximated depth profile of a cellulosic fiber.

TGA and FTIR analyses revealed that the CP fibers are mainly composed of cellulose, hemicellulose, and lignin. XRD and FTIR data demonstrated that the cellulose is anisotropic with a preferential direction for the alignment of the crystalline structures within cellulose and a variation in chemical composition from the surface to the interior of the fibers. FTIR data have also revealed that the mineral oil can be better adsorbed at the interior surface of their hollow tube structures, where there is a higher concentration of hemicellulose and probably lignin than at the external surface. Despite the higher oil adsorption in the interior surfaces, FTIR spectroscopy has indicated that oil was also adsorbed inside the tubular walls. Furthermore, there is strong evidence that the adsorption process is physisorption.

Regarding the filtration experiments, introducing 200 mg of these hydrophobic, oleophilic, tubular fibers (average diameter of 25 μm) into a cylindrical filter enabled, the removal of up to 67% of the mineral oil from an initial concentration of 145 mg L^–1^ for 250 mL of emulsion processed at a flow rate of 172 mL min^–1^. Notably, these CP fibers exhibited reusability, as they could be pressed to expel the absorbed oil and employed for up to three consecutive cycles in a filtration process. Even after the second compression, they remain highly effective, consistently eliminating more than 50% of the oil from the emulsion.

On the other hand, when the fibers were arranged in a semispherical geometry and forcibly submerged, they absorbed more than 95% of the emulsified oil in less than 5 min under agitation. There must be relative movement between the fibers and the fluid for significant absorption to occur; thus, the kinetic energy of the oil droplets plays a crucial role. Consequently, since most of the oil retention occurs at the internal surface, filtration system designs that force the fluid to flow inside the fibers will result in even higher oil removal from an effluent than what was observed up to date.

This work makes a significant contribution to the literature by providing a comprehensive characterization of natural C. procera fibers, detailing the distribution of their organic components (hemicellulose, lignin, cellulose and wax), and systematically evaluating the CP fibers̀ performance in a filtration process for oil removal from emulsions.

## Supplementary Material







## Notes

The support file contains five tables, nine figures and the deduction of eq 4. Table S1 describes the number of filtration experiments performed, Figure S1 shows details of the filtering system, Figure S2 contains a histogram detailing the range of diameters of the CP fibers, Figure S3: SEM micrographs used to measure CP fibers’ diameters, Figure S4: X-ray diffractograms (XRD) patterns for the CP fibers, Table S2 shows the assignment of the diffraction peaks, Table S3 compares the TGA results for C. procera fibers with previously reported data in the literature, Figure S5: TGA of Calotropis procera fibers harvested in December of 2022 (the fibers used in this work) and of fibers harvested in August of 2023, Figure S6: FTIR of Calotropis procera fibers harvested in December of 2022 (the fibers used in this work) and of fibers harvested in August of 2023, Table S4: FTIR band assignments and intensities for CP fibers, Figure S7: Contact angle measurements, Figure S8: Sequential images illustrating the oil absorption process by a bed of C. procera fibers, Table S5: Spearman correlation coefficients between input variables and OR % for the cylindrical filtering system, Figure S9: Principal component analysis (PDF)
Water repulsion inside the CP fiber is observable (AVI)
The fibers in the oil either sink or remain submerged at what appear to be a constant depth due to the presence of air pockets (AVI)
