# Supplementary material for: Characterization and Application of Calotropis Procera Fiber as a Sustainable Filter for Oil Removal from Aqueous Emulsion
Source: ACS Omega. 2025 May 5;10(19):19655–64. doi: 10.1021/acsomega.5c00525 (PMC12096230; doi:10.1021/acsomega.5c00525)
Supplement: Supplementary file 1 [file ao5c00525_si_001.pdf]

Supporting Information for the Article: **Characterization and Application of *Calotropis Procera* Fiber as a Sustainable Filter for Oil Removal from Aqueous Emulsion**

Eduardo Perini Muniz[1*], Lucas Prandi Coutinho[1], Odilon Junior Gonçalves de Oliveira[1], Marla Almeida Siqueira[2], Paulo Sérgio da Silva Porto[1], Edson Caetano Passamani [3], José Rafael Capua Proveti[1], Cleocir José Dalmaschio[2].

[1] Programa de Pós-Graduação em Energia, Universidade Federal do Espírito Santo, Rodovia Governador Mario Covas, km 60, Bairro Litorâneo, São Mateus, ES, 29932-540, Brazil

[2] LabPetro - Programa de Pós-Graduação em Química, Universidade Federal do Espírito Santo, Av. Fernando Ferrari, 514 - Goiabeiras, Vitória – ES, 29075-910, Brazil

[3] Programa de Pós-Graduação em Física, Universidade Federal do Espírito Santo, Av. Fernando Ferrari, 514 - Goiabeiras, Vitória – ES, 29075-910, Brazil

*Corresponding author: e-mail eduardo.muniz@ufes.br

Table S.1 – Number of filtration experiments performed for each set of variables (Q is the flow rate (Q), and the mass of fibers used (MF))*.

FM (mg) \ Q ($mL.min^{-1}$)	88	130	172
25	1	1	1
50	1	1	1
100	**2**	**3**	**3**
200	**3**	**4**	**3**
300	**1**	**1**	**2**
1000			1

*The experiments marked in **bold** constitute a 3^2 full factorial experimental matrix that is the core of the experimental design used. The numbers in the body of the table indicate the replicates of the experiments.

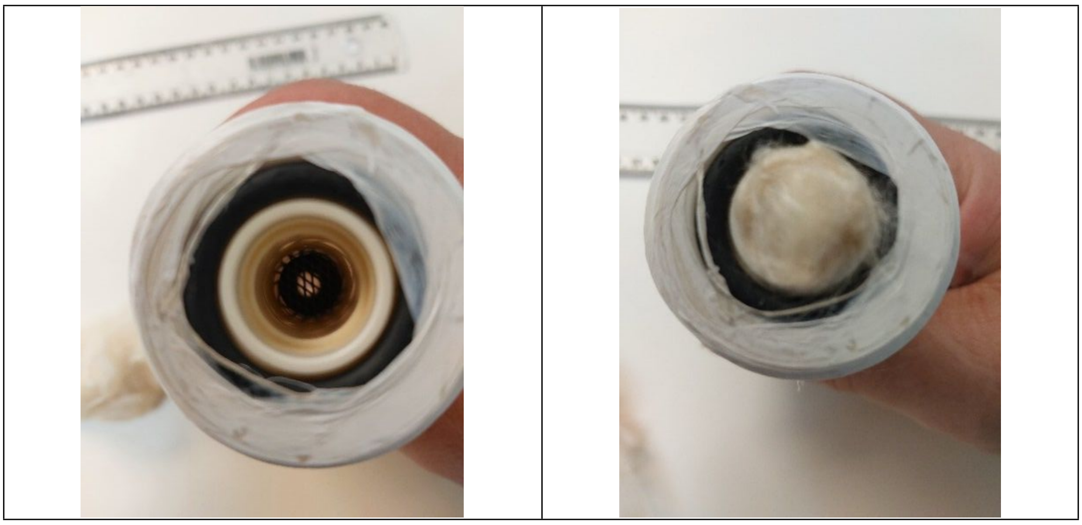

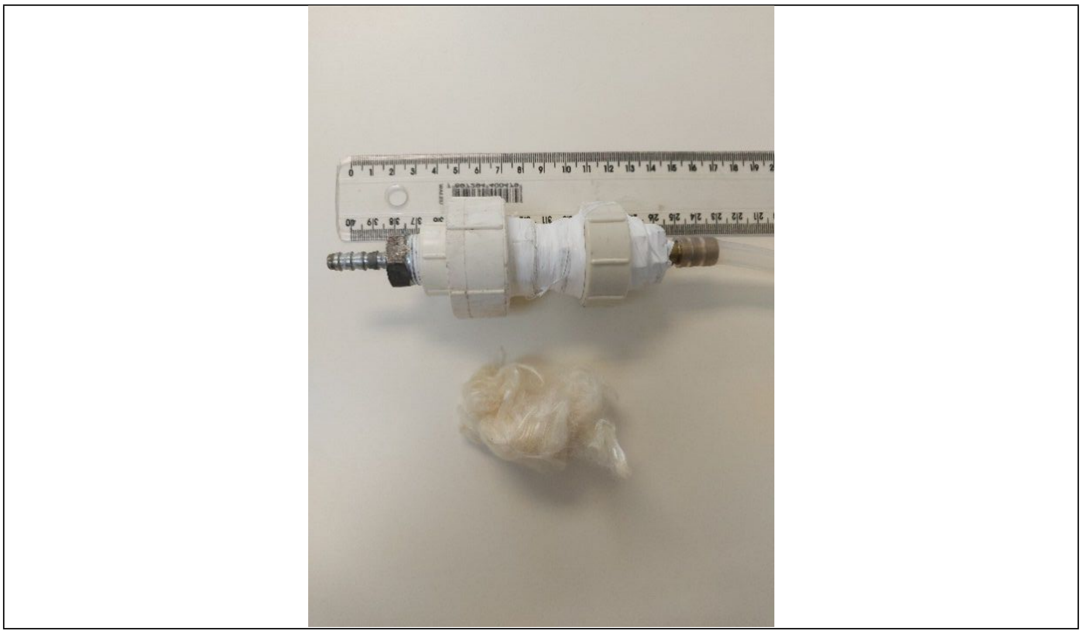

Figure S1: Details of the placement of the fiber bundle inside the filtering system.

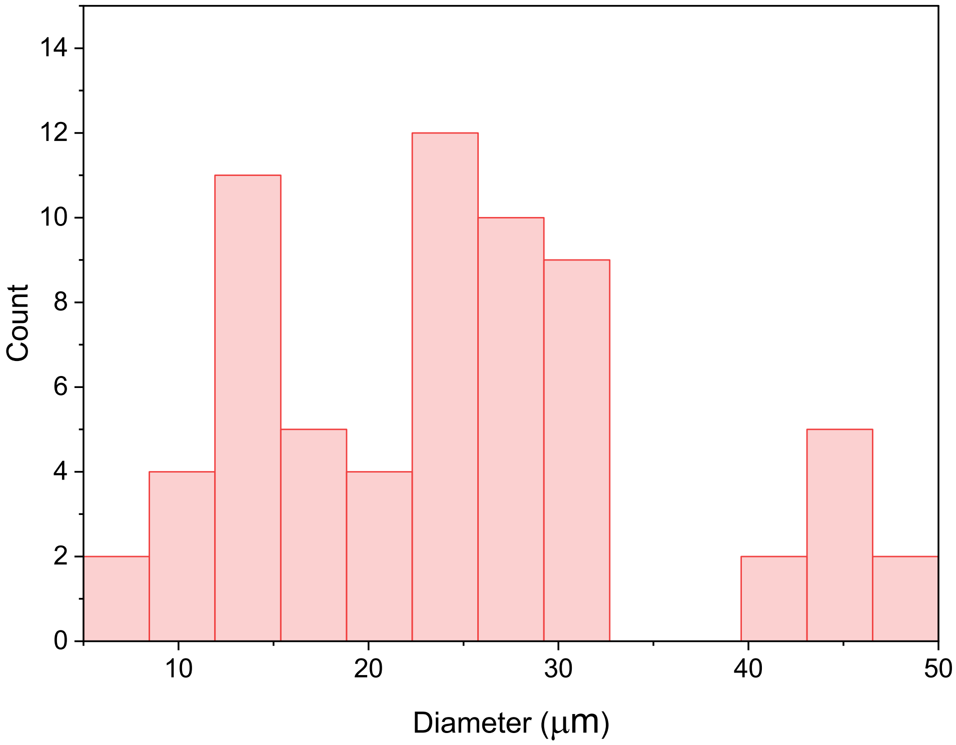

Figure S2: Histogram detailing the range of diameters of the CP fibers.

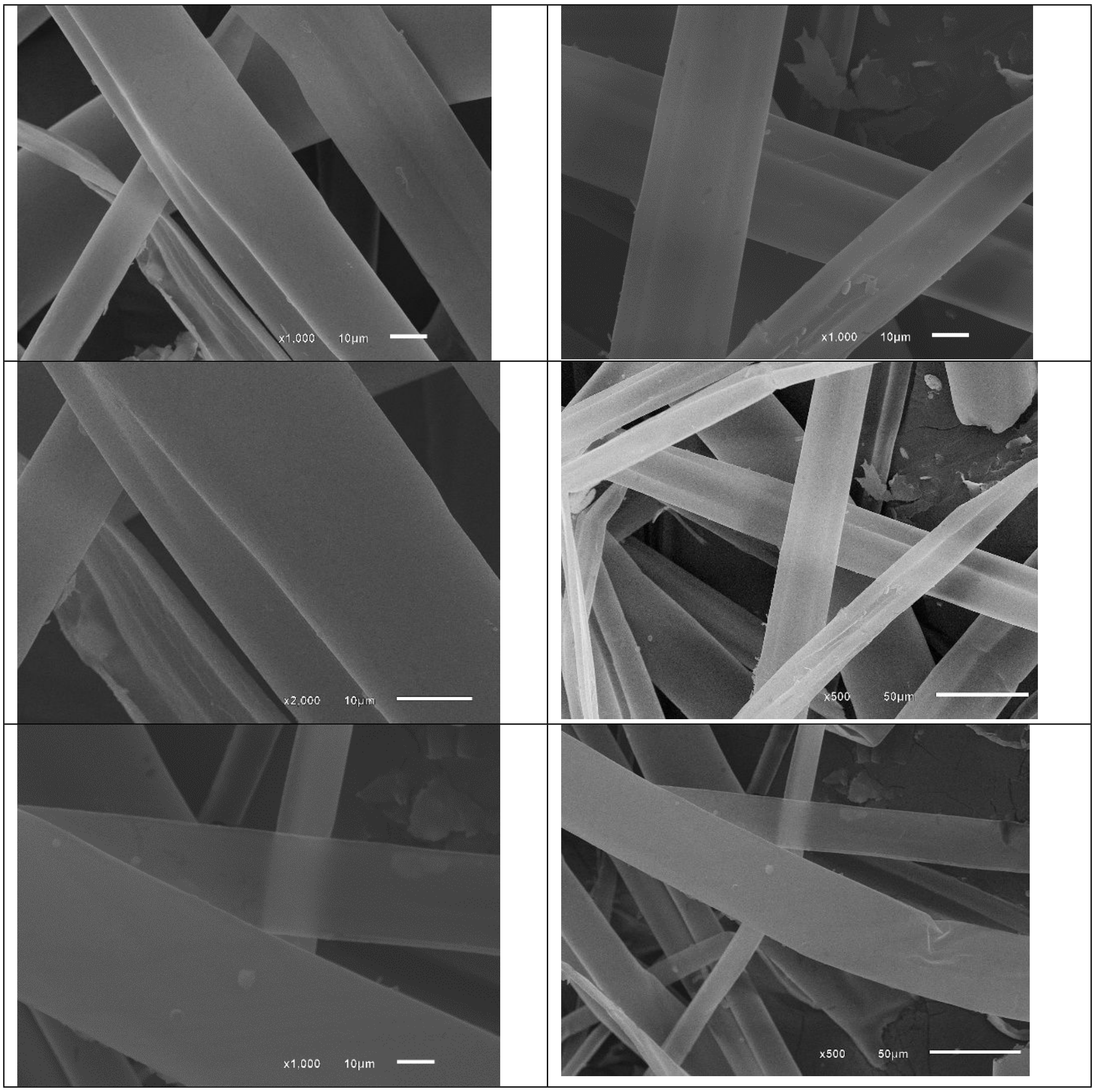

Figure S3: SEM micrographs used to measure CP fibers' diameters.

a)	b)

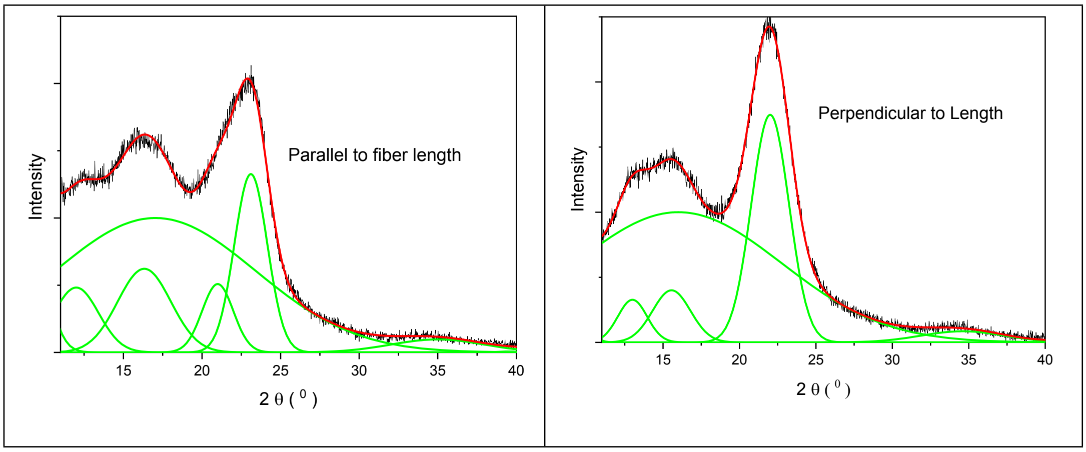

Figure S4 – X-ray diffractograms (XRD) patterns for the CP fibers a) with the incident X-ray beam aligned in the plane parallel to the fiber length and b) with the beam aligned in the plane perpendicular to the fiber length.

Table S2 – Assignment of the diffraction peaks.

**PEAK POSITION**		
**XRD parallel to fiber length (^{0})**	**XRD perpendicular to fiber length (^{0})**	**ASSIGNMENT**
**12.0**	**13.0**	(100) in cellulose I α and (1$\bar{1}$0) in cellulose I β [42].
**16.3**	**15.6**	(010) in cellulose I α and (110) in cellulose I β [42].
**21.0**		Superposition of peaks from cellulose I α and cellulose I β [42].
**23.1**	**22.0**	(110) in cellulose I α and (200) in cellulose I β [42].

**35.0**	**34.6**	(004) cellulose I β, superposition of peaks of cellulose I α [42].
**17.0**	**16.0**	Amorphous peak* [43]

*Since the sample was not treated to eliminate the other components, the amorphous peak is not only due to the amorphous part of the cellulose, it includes contributions from lignin and hemicellulose.

Table S3 – Comparison of thermogravimetric analysis (TGA) results for *Calotropis procera* fibers with previously reported data in the literature.

**REDUCTION IN RM% (%)**	**THIS WORK**	**HILÁRIO AND COWORKERS [1]**	**YOGANANDAM AND COWORKERS [2] ***	**MOSTLY ASSOCIATED WITH**
**FIRST EVENT**	7	5.9	7.457	Loss of moisture and water content
**SECOND EVENT**	57	58.8	47.093	Degradation of hemicellulose
**THIRD EVENT**	24	20	22.114	Degradation of Cellulose
**FOURTH EVENT**	7	9.76	3.888	Thermal decomposition of lignin

*CP fibers extracted from a branch.

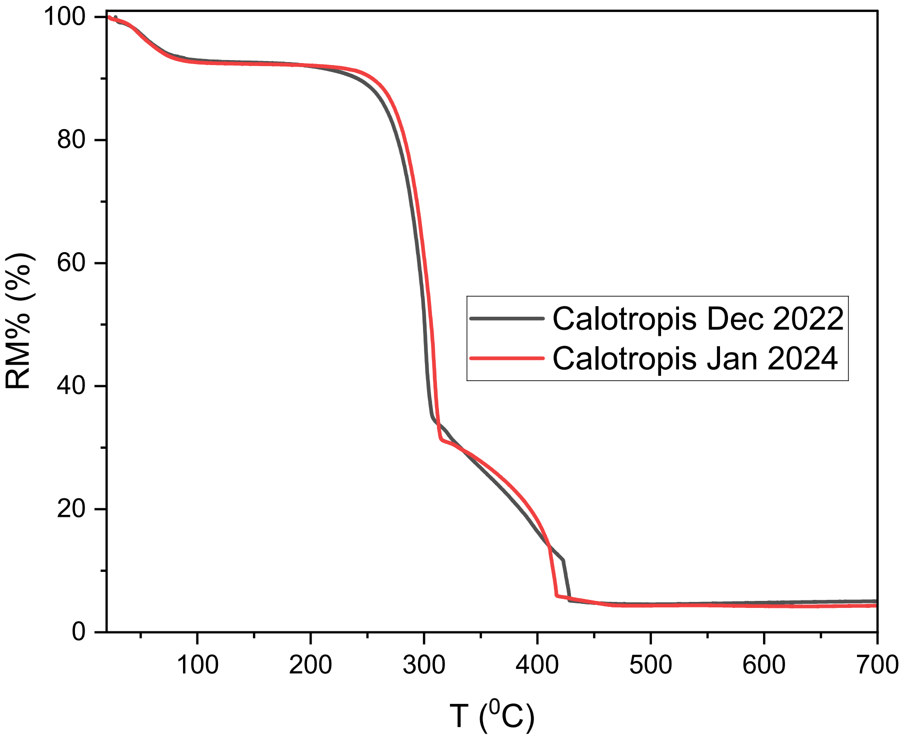

Figure S5: TGA of Calotorpis procera fibers harvested in December of 2022 (the fibers used in this work) and of fibers harvested in August of 2023.

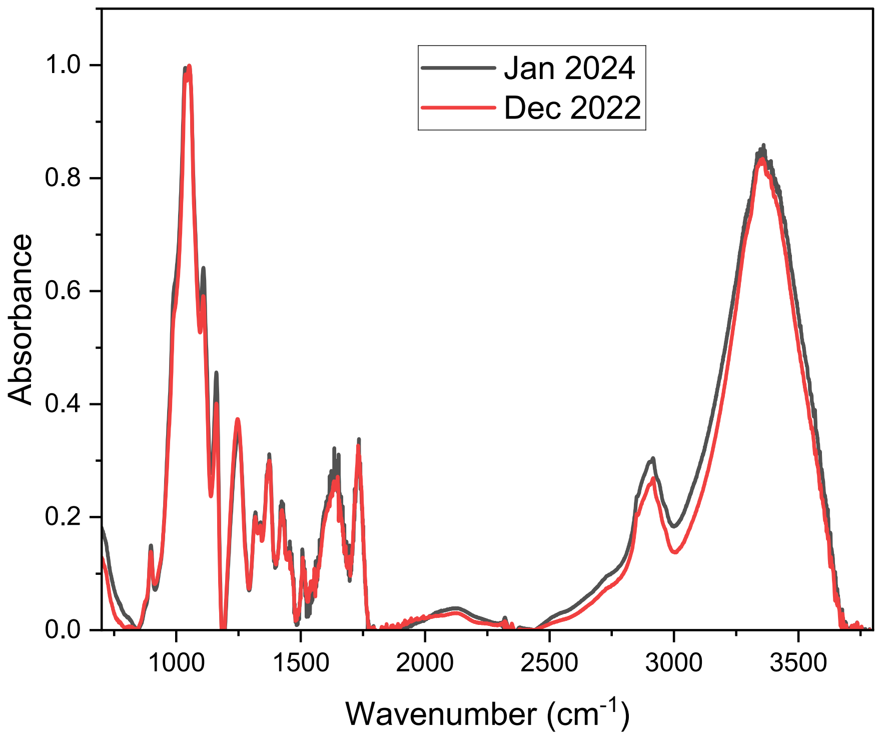

Figure S6: FTIR of Calotorpis procera fibers harvested in December of 2022 (the fibers used in this work) and of fibers harvested in August of 2023.

Table S4 - FTIR band assignments and intensities for CP fibers.

Wavenumber ($\pm$ 3 cm^{-1})	ATRG	ATRD	DPT	BAND ASSIGNMENTS	COMPONENT
**873**	0.05	0.05	0.07	δ C2-H [3]	Hemicellulose
**898**	0.12	0.11	0.17	Aromatic ring stretching vibration at the glycosidic linkage [3–5].<br>ω CH_2 in alkanes [6]	Cellulose<br>Hemicellulose<br>Alkanes in Wax
**990**	0.62	0.62	0.62	C-O Valence vibration [7]	-
**1033**	1	1	1	ν C6-O6H in cellulose [3,5]<br>ν C-O in lignin (guaiacyl ring) [5]	Cellulose<br>Lignin
**1051**	**0.86**	0.83	1	ν C3-O3H [3]	Cellulose<br>Hemicellulose
**1107**	0.40	0.36	0.64	ν C2-O2H [3]<br>Aromatic<br>Ring stretching [6,8]	Cellulose I<br>Hemicellulose<br>Wax
**1160**	0.21	0.17	0.44	ν C1-O-C4 [3,5,8].<br>ν C-O in alcohol [6]	Cellulose I<br>Hemicellulose<br>Alcohol structure in wax.

**1244**	0.27	0.24	0.43	ν C-O in a carboxylic acid [3], syringly ring breathing, and in ν C-O in lignin [7]	Hemicellulose Lignin
**1317**	0.12	0.09	0.20	δ O-H in the plane [3], C – O of syringyl ring in Lignin [5], ω CH_2 in cellulose [5,7].	Cellulose Hemicellulose Lignin
**1336**	0.11	0.07	0.18	δ O-H in plane in cellulose [3,5] δ C-H in Lignin [3]	Cellulose Lignin
**1362**	0.15	0.12		δ C-H in cellulose II [8]	Cellulose II
**1369**	0.16	0.13		δ C-H_3 [9]	Cellulose
**1374**	0.18	0.14	0.32	δ C-H [3,5] CH_3 symmetric deformation; OH deformation of carboxyl monomer [6]	Cellulose Hemicellulose Wax
**1386**	0.10	0.07		ν COO^- [3]	Hemicellulose
**1419**	0.12	0.09	0.24	Aliphatic C-H [10]	Cellulose
**1425**	0.11	0.09	0.27	δ O-H in plane δ C-H in CH_3 [3], with aromatic ring stretching in Lignin [5]	Cellulose I Hemicellulose Lignin

				δ CH_2 in cellulose I [8]	
**1428**	0.11	0.09		δ C-H [5]	Cellulose
**1435**	0.09	0.08		δ C-H_2 or δ C-H_3	Wax [6]
**1449**	0.08	0.06	0.13	δ O-H in plane [3]	Cellulose and Hemicellulose
**1456**	0.10	0.07		δ C-H in CH_2 and CH_3 [9]	Cellulose and Hemicellulose
**1464**	0.05	0.05	0.16	δ C-H in CH_2 and CH_3 (lignin) [3,5]<br>Scis C-H_2 [6]	Lignin<br>Wax
**1472**	0.03	0.03		Scis C-H_2 [6]	Wax
**1508**	0.08	0.08	0.16	Aromatic skeletal vibration [3,5,9]	Lignin<br>Cellulose
**1633**	0.13	0.13	0.23	ν C=O [10,11]<br>δ O-H in water	Cellulose<br>Water
**1732**	0.21	0.18	0.37	ν C=O [3]	Hemicellulose
**2850**	0.09	0.13	0.22	ν C-H	
**2898**	0.11	0.15	0.28	ν C-H	
**2916**	0.10	0.15	0.28	ν C-H [4]	Cellulose
**2940**	0.07		0.24	ν C-H [5]	Lignin
			0.20	ν C-H	
**3335**	0.28	0.32	0.79	ν O-H	

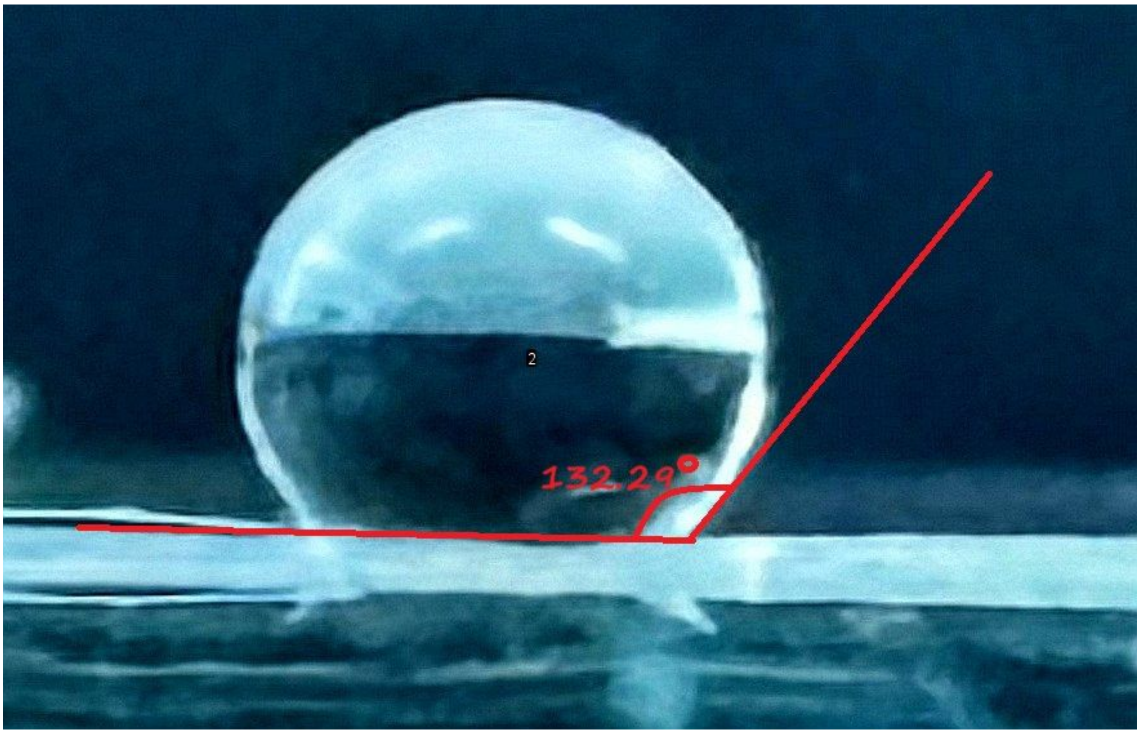

Figure S7 – Contact angle measurement of water on the surface of the white layer of *Calotropis procera* fibers arranged as a fiber bed, demonstrating the material's hydrophilic or hydrophobic properties.

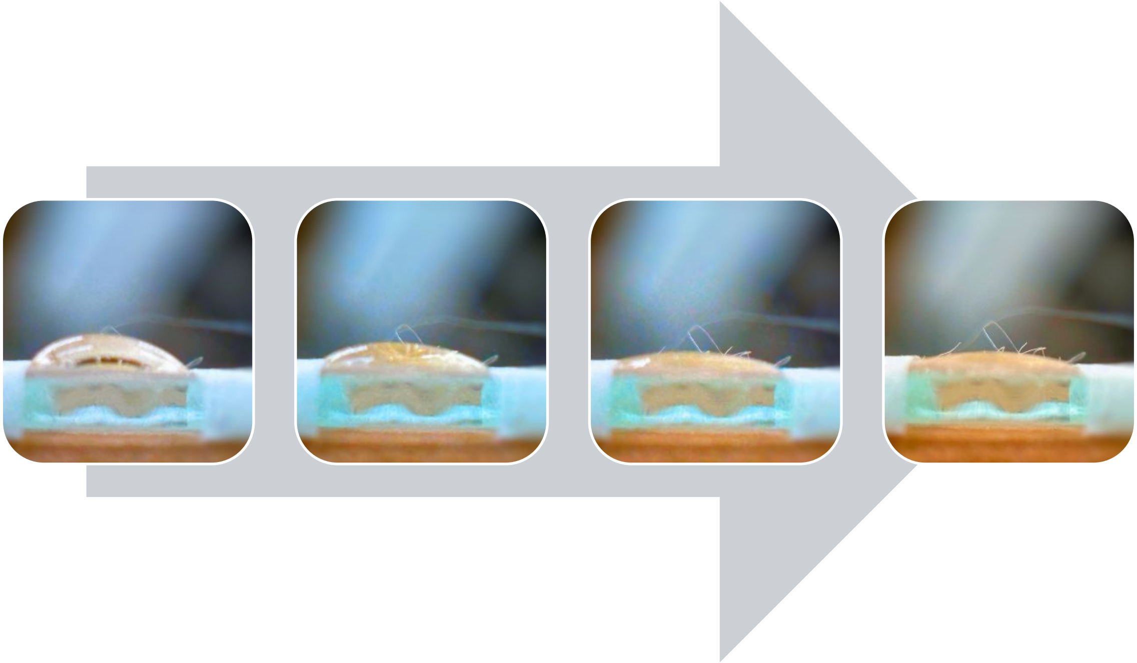

Figure S8 – Sequential images illustrating the oil absorption process by a bed of *Calotropis procera* fibers, captured at 20-second intervals over a total duration of approximately 1 minute.

Table S5 - Spearman correlations coefficient between input variables and OR% for the cylindrical filtering system.

Input Variable	Spearman Correlation Coefficient with OR%	p-value
Q	0.23573	0.33128
FM	0.94498	< 0.0001

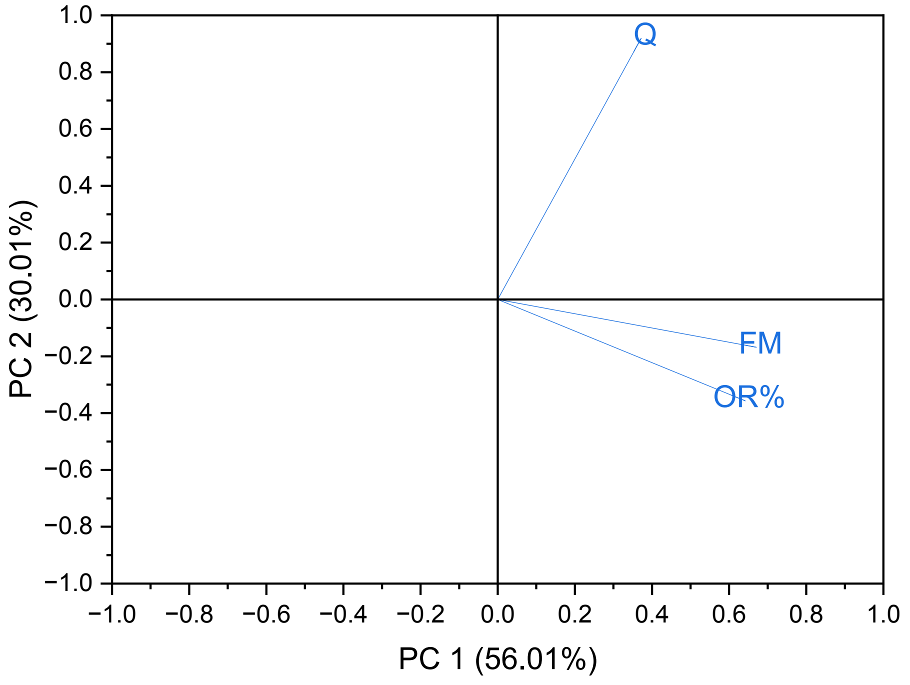

Figure S9 – Principal component analysis.

Mathematical deduction of Eq. 4

Eq. (4) can be derived if k, the ratio of oil removal per length of filter, is constant along the length of the CP filter and with time. If these two hypotheses are valid, Eq. (S1) applies to the concentration C_l of oil along the filter.

$$\frac{dC_l}{dl} = -kC_l \qquad \text{(S1)}$$

Solving Eq. (S1), substituting the result in Eq. (4), and applying the conditions that OR% = ORf% when l -> ∞ and OR% = 0 if l = 0 results in Eq. (S2).

$$OR\% = ORf\%(1 - e^{-kl}) \qquad \text{(S2)}$$

The contour condition OR% = 0 when l = 0 can be applied since C_i in Eq. (3) is the concentration of oil in the effluent after it went through the system in the absence of CP fibers. It includes any eventual reduction in concentration due to the flow of the effluent through the gratings and tubes.

Eq. (S2) is like that one given by Davies in 1974 for the retention of particles by fibrous filters in air [12]. The main difference is that the equation derived in 1974 converges to 100% removal of particles when the filter has an infinite length, whereas Eq. (S2) considers that there is a limit to the amount of oil that can be removed.

To go from Eq. (S2) to Eq. (4), it must be considered that the length of the filtrating element increases with FM. The length of the tube was not small enough to compress the fibers, but the diameter was (see Fig. S1). Thus, the fiber net always filled the full diameter of the tube, but the length of occupation depended on the fiber mass. If the volume of the fibers for a given tube volume (i.e., the volumetric packing factor, c) is constant, the Eq. (S3) can be derived.

$$MF = c.\rho.\pi.R^2.l \qquad \text{(S3)}$$

From Eqs.(S3) and (S2), Eq. (4) can be obtained if B = k/(c.$\rho.\pi.R^2$) where R is the radius of the tube (0.75 cm, see Fig. 1), ρ is the density of the CP fibers.

Bibliography

(1) Sobral Hilário, L.; Batista dos Anjos, R.; Borges de Moraes Juviniano, H.; Ribeiro da Silva, D. Evaluation of Thermally Treated Calotropis Procera Fiber for the Removal of Crude Oil on the Water Surface. *Materials (Basel)* **2019**, *12* (23), 3894. https://doi.org/10.3390/ma12233894.

(2) Yoganandam, K.; NagarajaGanesh, B.; Ganeshan, P.; Raja, K. Thermogravimetric Analysis of Calotropis Procera Fibers and Their Influence on the Thermal Conductivity and Flammability Studies of Polymer Composites. *Mater. Res. Express* **2019**, *6* (10), 105341. https://doi.org/10.1088/2053-1591/ab3bbe.

(3) Horikawa, Y.; Hirano, S.; Mihashi, A.; Kobayashi, Y.; Zhai, S.; Sugiyama, J. Prediction of Lignin Contents from Infrared Spectroscopy: Chemical Digestion and Lignin/Biomass Ratios of Cryptomeria Japonica. *Appl Biochem Biotechnol* **2019**, *188* (4), 1066–1076. https://doi.org/10.1007/s12010-019-02965-8.

(4) Chen, W.; He, H.; Zhu, H.; Cheng, M.; Li, Y.; Wang, S. Thermo-Responsive Cellulose-Based Material with Switchable Wettability for Controllable Oil/Water Separation. *Polymers* **2018**, *10* (6), 592. https://doi.org/10.3390/polym10060592.

(5) Pandey, K. K. A Study of Chemical Structure of Soft and Hardwood and Wood Polymers by FTIR Spectroscopy. *Journal of Applied Polymer Science* **1999**, *71* (12), 1969–1975. https://doi.org/10.1002/(SICI)1097-4628(19990321)71:12<1969::AID-APP6>3.0.CO;2-D.

(6) Farber, C.; Li, J.; Hager, E.; Chemelewski, R.; Mullet, J.; Rogachev, A. Yu.; Kurouski, D. Complementarity of Raman and Infrared Spectroscopy for Structural Characterization of Plant Epicuticular Waxes. *ACS Omega* **2019**, *4* (2), 3700–3707. https://doi.org/10.1021/acsomega.8b03675.

(7) El Oudiani, A.; Msahli, S.; Sakli, F. In-Depth Study of Agave Fiber Structure Using Fourier Transform Infrared Spectroscopy. *Carbohydrate Polymers* **2017**, *164*, 242–248. https://doi.org/10.1016/j.carbpol.2017.01.091.

(8) Yang, Y. P.; Zhang, Y.; Lang, Y. X.; Yu, M. H. Structural ATR-IR Analysis of Cellulose Fibers Prepared from a NaOH Complex Aqueous Solution. *IOP Conf. Ser.: Mater. Sci. Eng.* **2017**, *213* (1), 012039. https://doi.org/10.1088/1757-899X/213/1/012039.

(9) Li, X.; Wei, Y.; Xu, J.; Xu, N.; He, Y. Quantitative Visualization of Lignocellulose Components in Transverse Sections of Moso Bamboo Based on FTIR Macro- and Micro-Spectroscopy Coupled with Chemometrics. *Biotechnology for Biofuels* **2018**, *11* (1), 263. https://doi.org/10.1186/s13068-018-1251-4.

(10) Jackson, M. J.; Line, M. A. Organic Composition of a Pulp and Paper Mill Sludge Determined by FTIR, 13C CP MAS NMR, and Chemical Extraction Techniques. *J. Agric. Food Chem.* **1997**, *45* (6), 2354–2358. https://doi.org/10.1021/jf960946l.

(11) Nor Fazelin, M. Z.; Yusop, S. M.; Ahmad, I. Preparation and Characterization of Cellulose and Nanocellulose from Pomelo (Citrus Grandis) Albedo. *Journal of Nutrition and Food Sciences* **2015**, *5* (1).

(12) Davies, C. N. The Retention of Particles in Filters. *Journal of Aerosol Science* **1974**, *5* (5), 487–495. https://doi.org/10.1016/0021-8502(74)90089-5.
